# Nivolumab-Induced, Late-Onset, Steroid-Sensitive, High-Grade Pneumonitis and Durable Tumor Suppression in Metastatic Renal Cell Carcinoma: A Case Report

**DOI:** 10.1155/2019/6759472

**Published:** 2019-12-26

**Authors:** Vincent Louie Mendiola, Meghana Kesireddy, Bagi Jana

**Affiliations:** ^1^Department of Internal Medicine, University of Texas Medical Branch, Galveston, TX, USA; ^2^Hematology/Oncology, University of Texas Medical Branch, Galveston, TX, USA

## Abstract

Nivolumab, an antiprogrammed death-1 checkpoint inhibitor, has been approved for use in unresectable/metastatic renal cell carcinoma (RCC). Nivolumab-induced pneumonitis, a rare, but often severe and potentially life-threatening immune-related adverse event, has been reported, typically, early during the treatment. Due to its low incidence, more studies are needed to better elucidate this condition and its possible effects on cancer progression. We now present a 57-year-old Hispanic male patient with metastatic RCC-clear cell type who, after his 34^th^ cycle of nivolumab (16 months after being on nivolumab), developed a late-onset, immune-related adverse event (IRAE) including a grade 3 pneumonitis, which resolved completely, clinically, and on serial lung imaging with steroids and drug discontinuation. His cancer remained stable with no progression for 18 months despite discontinuation of nivolumab which showed tumor progression resistance. This case report is aimed at providing further information regarding the rare phenomena of a late-onset IRAE, in particular, a grade 3 nivolumab-induced pneumonitis which also responded rapidly to treatment, as well as at discussing this immunotherapy's durable tumor suppressive effect and a possible associated factor to this phenomenon.

## 1. Background

RCC-clear cell (CC) type is the most common type of kidney cancer among adults with a global incidence of about 337,000 cases and 143,000 deaths annually [1].

Nivolumab, a monoclonal antibody that selectively inhibits programmed cell death-1 (PD1) activity, is approved for patients with metastatic/unresectable renal cell carcinoma (RCC) who failed prior antiangiogenic therapy. Pneumonitis, a rare immune-related adverse event (IRAE) that is life-threatening, occurred in 5% of 406 patients [2–4] and the median duration of treatment before pneumonitis onset is typically 2.8 months [5].

## 2. Case Presentation

**Figure 1:**
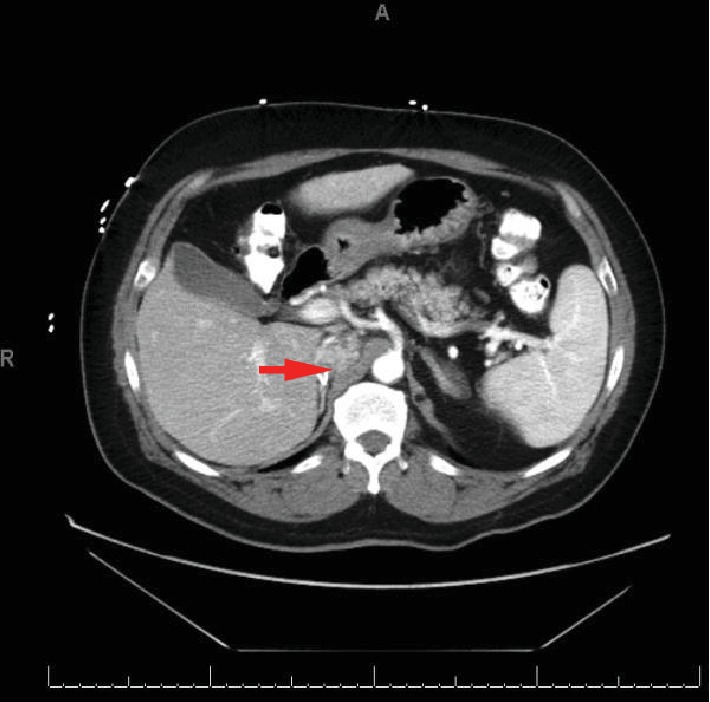
CT abdomen and pelvis w/ contrast; arrow showing heterogenous density 2.5 × 3.5 cm lesion in vicinity of the right nephrectomy bed compressing the inferior vena cava.

**Figure 2:**
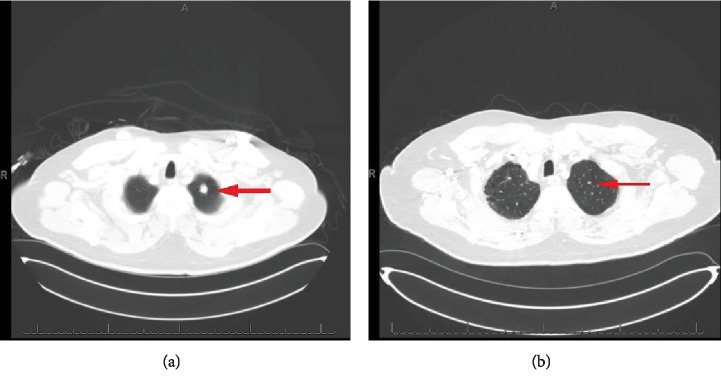
(a) CT chest w/ contrast. Arrow showing a 1.4 cm peripherally enhancing, partially speculated nodule in the left upper lung apex. (b) CT chest w/ contrast; ~18 months post discontinuation of nivolumab with the following findings: arrow showing a 0.4 cm enhancing nodule in the left upper lung lobe.

A 57-year-old Hispanic male was diagnosed with RCC-CC type and underwent radical nephrectomy. His RCC was staged as stage III (pT3N0M0) and graded as Fuhrman grade 3. He was lost to follow-up, and four years later, he was found to have a soft tissue mass at the nephrectomy surgical bed, indicating local cancer recurrence (Figure 1). This, along with multiple spiculated nodules in the right and left lung, is the largest being 1.4 cm (Figure 2(a)). MRI brain and bone scan were negative. Fine needle aspiration of the lung nodule confirmed metastatic RCC-clear cell type. His international metastatic RCC database consortium (IMDC) score at representation was 2-3 (patient could have had metastases >1 year before representation for systemic therapy as he was lost to follow-up for years, Karnofsky <80%, hemoglobin less than normal, while his calcium levels, neutrophil, and platelet counts were low-normal), suggesting intermediate to poor risk of mortality, with a median survival of 7.8-22.5 months.

He was initially started on antiangiogenic agents, pazopanib followed by everolimus, which he did not tolerate due to side effects of vomiting, diarrhea, mouth ulcers, and palmar-plantar erythrodysesthesia. He was then treated with bevacizumab plus interferon alfa-2b but had cancer progression on this therapy. Finally, nivolumab (3 mg/kg, q2 weekly cycles) was started which he tolerated well, and surveillance imaging showed stable pulmonary nodules with a decrease in the size of the tumor at his nephrectomy site.

**Figure 3:**
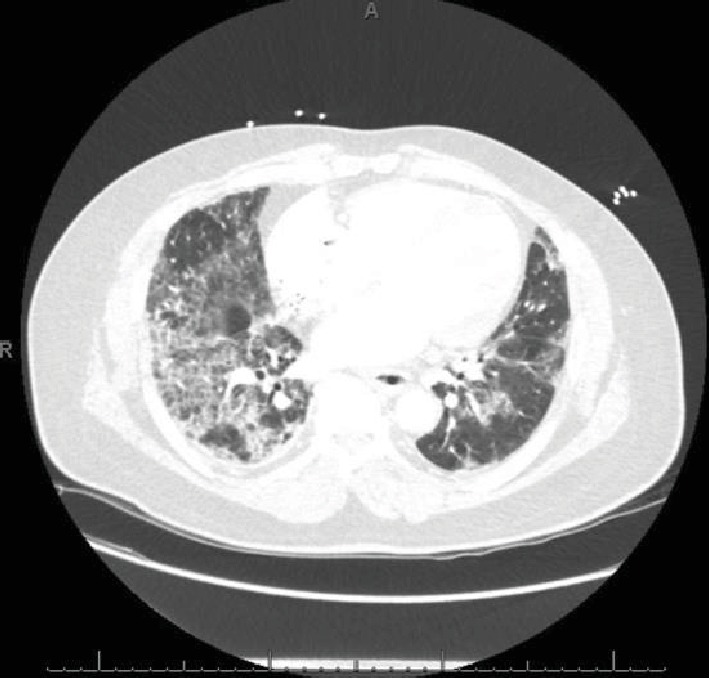
CT chest w/ contrast, lung window, showing initial acute pneumonitis findings: extensive ground-glass opacities and interlobular septal thickening with bilateral traction bronchiectasis. Pulmonary nodules are not well seen due to ground-glass opacities.

Seventeen days after his 34^th^ cycle (after 16 months), he presented to the clinic with a 10-day history of productive cough (clear sputum) and shortness of breath. While being evaluated for these respiratory symptoms in the outpatient setting, in a span of 2 days, he developed a rash on his bilateral palms and soles and got admitted to the hospital for acute hypoxic respiratory failure (a respiratory rate of 30, O_2_ saturation of 81 on room air, partial O_2_ of 57 on arterial blood gas) requiring a nonrebreather mask (with 100% fraction of inspired oxygen (FiO_2_) on 15 L/minute flow). Physical examination revealed bilateral diffuse crackles and thick plaques/callouses of his bilateral palms and soles. CT chest showed confluent ground-glass and reticular opacities in bilateral lungs predominantly in the bases (Figure 3) concerning for pneumonitis from nivolumab therapy. Follow-up serologic tests, sputum, and blood cultures were negative, and bronchoscopy was not done due to suggestive findings on imaging.

He was started on high-dose steroids, methylprednisolone IV 2 mg/kg (160 mg) daily, which resulted in significant improvement of his cough, dyspnea, hypoxia, and skin rash in a couple of days. After five days of IV steroids, he was switched to prednisone 40 mg oral daily. On day ten of steroids, he was successfully weaned off supplemental oxygen and discharged with a prolonged prednisone taper of 40 mg PO daily for two weeks followed by 20 mg PO daily for two weeks, and then 10 mg PO daily for one week. Nivolumab was discontinued and held indefinitely.

**Figure 4:**
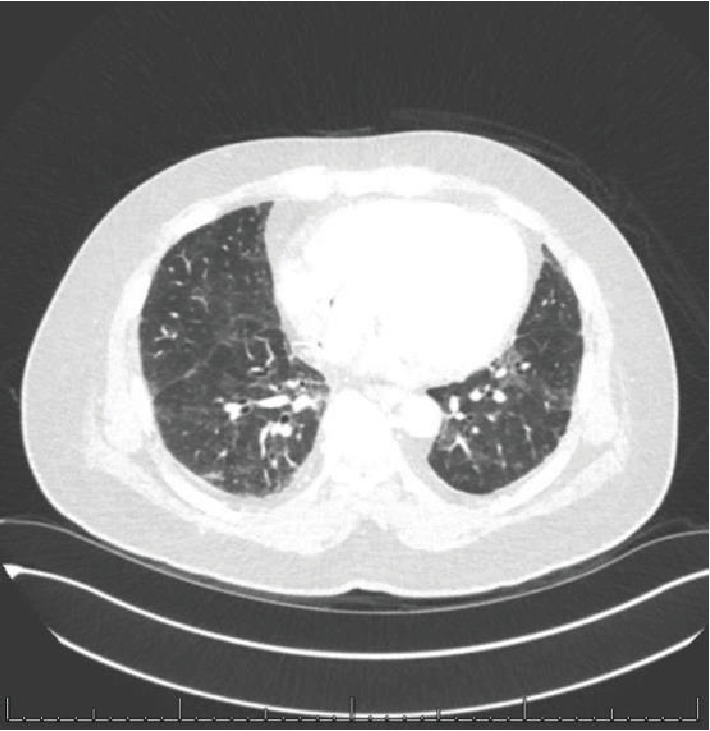
CT chest w/ contrast, lung window, showing lung findings post ~1.5 months of initial pneumonitis findings: significant improvement in scattered interstitial thickening and ground-glass opacities.

**Figure 5:**
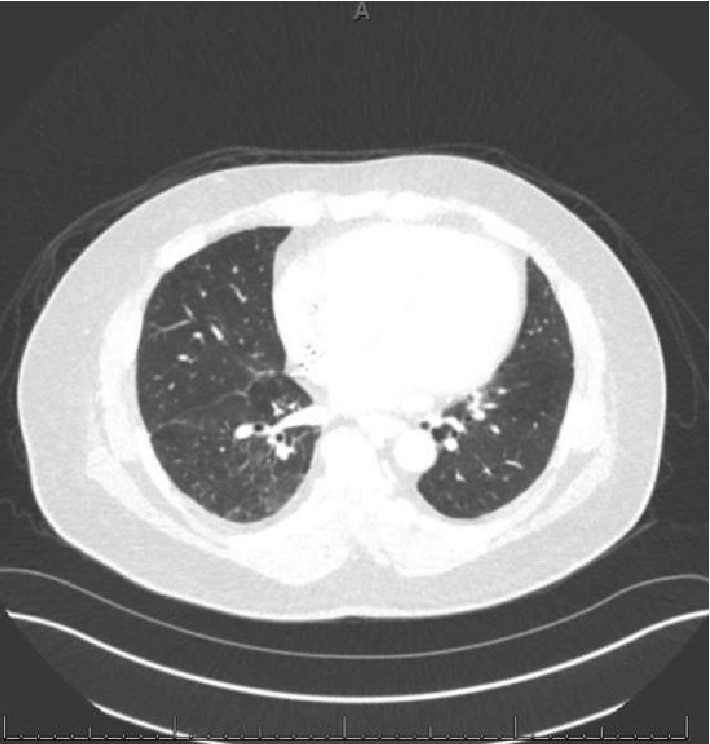
CT chest w/ contrast, lung window, showing lung findings post ~4 months of initial pneumonitis findings: mild emphysematous changes noted.

A CT chest obtained 1.5 months after his hospital discharge showed significantly improved ground-glass opacities in both lungs, while his pulmonary nodules remained unchanged (Figure 4). He was monitored closely with CT chest, abdomen, and pelvis, and after four months (post pneumonitis admission), the ground-glass opacities have improved significantly (Figure 5), along with stable pulmonary nodules, and a stable nephrectomy site mass that remained unchanged on serial imaging.

## 3. Discussion

Regarding our patient's pneumonitis onset, the typical median duration of treatment before pneumonitis was 2.6 months in non-RCC patients and 5.5 months in RCC patients [6–9]. Our patient developed pneumonitis along with synchronous dermatological manifestations 16 months after nivolumab initiation implying late-onset toxicity which is extremely rare. Our case report highlights that nivolumab-induced pneumonitis, an IRAE, can occur at any time after therapy initiation, even late in its treatment, and can co-occur with other IRAEs as well. The pathological mechanism involved and the factors predisposing to late-onset IRAE are yet to be determined. In addition, due to this phenomenon's rarity, it is important to rule out any infectious etiologies including fungal or viral as a differential, especially in immunosuppressed patients, by using a combination of clinical judgement in terms of patient presentation, proximity of factors to inciting event, along with laboratory and radiographic tests such as serologic tests, cultures, and response to treatments. In our patient, cultures were negative, imaging showed the classic ground-glass opacities noted in pneumonitis features, and, although proximity to nivolumab treatment was atypical, our patient still improved with just steroids, with no antibiotics being given. He also did not have any aspiration risks putting chemical-induced pneumonitis lower on his differential as well.

Regarding his pneumonitis management, about 42% of nivolumab-induced grade 3/4 pneumonitis patients died despite therapy with steroids and immunosuppressants [5, 10]. Our patient's grade 3 pneumonitis and skin rash responded rapidly to high-dose steroids and completely resolved (both clinically and radiologically). This excellent steroid response in our patient could be associated with different pathological mechanisms involved in the development of late-onset IRAEs compared to the typical/early onset IRAEs and needs further elucidation.

**Figure 6:**
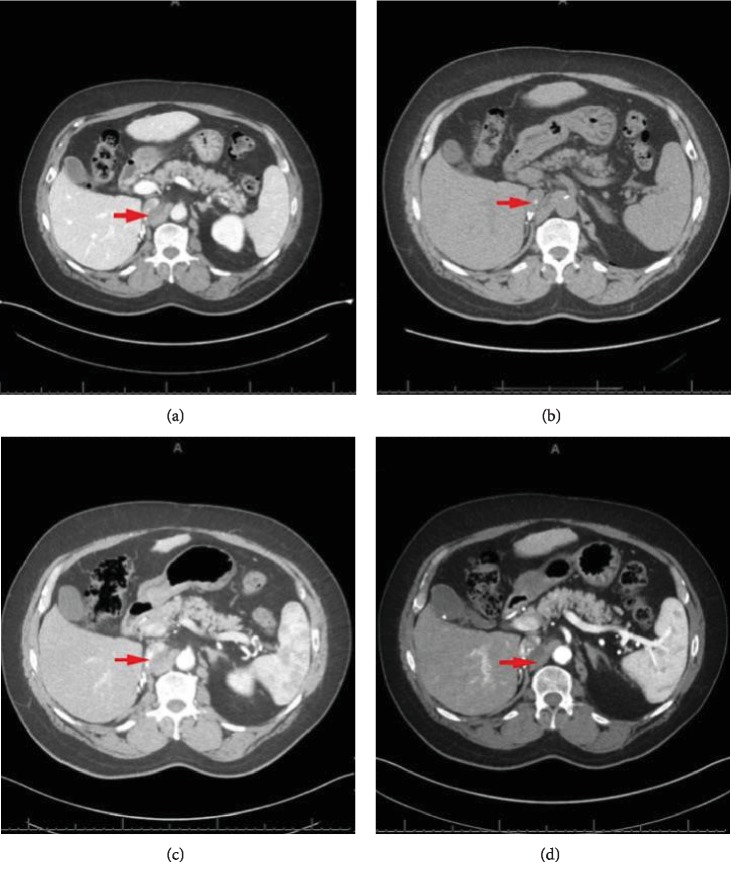
(a) CT abdomen and pelvis w/ contrast; ~1.5 months post discontinuation of nivolumab with the following findings: arrow showing a heterogenous density 3.5 × 1.0 cm lesion in the right retroperitoneal space unchanged from prior imaging (around time of acute pneumonitis occurrence). (b) CT abdomen and pelvis w/ contrast; ~4 months post discontinuation of nivolumab with the following findings: arrow showing a 3 × 1.0 cm soft tissue lesion posterior to inferior vena cava, stable in size. (c) CT abdomen and pelvis w/ contrast; ~9 months post discontinuation of nivolumab with the following findings: arrow showing a 3.1 × 1.3 cm soft tissue lesion posterior to inferior vena cava, stable in size. Of note, supra renal inferior vena cava tumor thrombus was noted measuring 2.8 × 1.5 cm. (d) CT abdomen and pelvis w/ contrast; ~18 months post discontinuation of nivolumab with the following findings: arrow showing a 2.8 × 1.5 cm soft tissue lesion posterior to inferior vena cava, stable in size. The supra renal inferior vena cava tumor thrombus measured at 2.1 × 1.4 cm, improved from prior imaging.

Regarding nivolumab therapeutic durability, Takagi et al. showed a durable response of 6 months after discontinuation of the drug before a metastatic RCC patient had disease progression [11]. Our patient had stable cancer burden with no progression for 18 months and ongoing, despite discontinuing nivolumab therapy (Figures 2(b) and 6(a)–6(d)). This therapeutic durability could be related to the following: the antitumor effect of the pathological immune mechanism responsible for his late-onset IRAE (pneumonitis in our case), from the additional antitumor effect of steroids used during his pneumonitis treatment or from the durable antitumor effect of nivolumab in and of itself.

Of note, the tumor suppressor effect of glucocorticoids has been elucidated in animal experimental models in the past and may be playing a crucial role in tumor suppression durability resulting to glucocorticoids being commonly used in chemotherapy regimens in hematologic and solid tumor malignancies [12–15]. In terms of our patient, more information is needed to elucidate if the rapid response of his rare, late-onset IRAE along with his ongoing stable metastatic cancer, off his nivolumab and any other current therapy, may be due to steroids in particular.

## 4. Conclusion

Further studies are necessary to understand the predisposing factors and pathological mechanisms involved in late-onset IRAE. In addition, further elucidation is needed to see if these mechanisms are also responsible for the excellent steroid response of this patient's late-onset IRAE or even the durable suppressive effect on metastatic RCC-CC tumor as seen in our patient.

## Abbreviations

RCC: Renal cell carcinoma
CC: Clear cell
RCT: Randomized control trial
OS: Overall survival
IRAE: Immune-related adverse event
IV: Intravenous
PO: Per oral
O_2_: Oxygen
FiO_2_: Fraction of inspired oxygen.
